# Airflow Dynamics of Human Jets: Sneezing and Breathing - Potential Sources of Infectious Aerosols

**DOI:** 10.1371/journal.pone.0059970

**Published:** 2013-04-01

**Authors:** Julian W. Tang, Andre D. Nicolle, Christian A. Klettner, Jovan Pantelic, Liangde Wang, Amin Bin Suhaimi, Ashlynn Y. L. Tan, Garrett W. X. Ong, Ruikun Su, Chandra Sekhar, David D. W. Cheong, Kwok Wai Tham

**Affiliations:** 1 Alberta Provincial Laboratory for Public Health, University of Alberta Hospital, Edmonton, Canada; 2 Department of Medical Microbiology and Immunology, University of Alberta, Edmonton, Canada; 3 Department of Laboratory Medicine, National University Hospital, Singapore, Singapore; 4 Department of Building, School of Design and Environment, National University of Singapore, Singapore, Singapore; Virginia Polytechnic Institute and State University, United States of America

## Abstract

Natural human exhalation flows such as coughing, sneezing and breathing can be considered as ‘jet-like’ airflows in the sense that they are produced from a single source in a single exhalation effort, with a relatively symmetrical, conical geometry. Although coughing and sneezing have garnered much attention as potential, explosive sources of infectious aerosols, these are relatively rare events during daily life, whereas breathing is necessary for life and is performed continuously. Real-time shadowgraph imaging was used to visualise and capture high-speed images of healthy volunteers sneezing and breathing (through the nose – nasally, and through the mouth - orally). Six volunteers, who were able to respond to the pepper sneeze stimulus, were recruited for the sneezing experiments (2 women: 27.5±6.36 years; 4 men: 29.25±10.53 years). The maximum visible distance over which the sneeze plumes (or puffs) travelled was 0.6 m, the maximum sneeze velocity derived from these measured distances was 4.5 m/s. The maximum 2-dimensional (2-D) area of dissemination of these sneezes was 0.2 m^2^. The corresponding derived parameter, the maximum 2-D area expansion rate of these sneezes was 2 m^2^/s. For nasal breathing, the maximum propagation distance and derived velocity were 0.6 m and 1.4 m/s, respectively. The maximum 2-D area of dissemination and derived expansion rate were 0.11 m^2^ and 0.16 m^2^/s, respectively. Similarly, for mouth breathing, the maximum propagation distance and derived velocity were 0.8 m and 1.3 m/s, respectively. The maximum 2-D area of dissemination and derived expansion rate were 0.18 m^2^ and 0.17 m^2^/s, respectively. Surprisingly, a comparison of the maximum exit velocities of sneezing reported here with those obtained from coughing (published previously) demonstrated that they are relatively similar, and not extremely high. This is in contrast with some earlier estimates of sneeze velocities, and some reasons for this difference are discussed.

## Introduction

Natural human exhalation flows such as coughing, sneezing and breathing can be considered as ‘jet-like’ airflows in the sense that they are produced from a single source in a single exhalation effort, with a relatively symmetrical, conical geometry. Although coughing and sneezing have garnered much attention as potential, explosive sources of infectious aerosols, these are relatively rare events during daily life, whereas breathing is necessary for life and is performed continuously. In the aftermath of the severe acute respiratory syndrome (SARS) outbreaks, the ongoing concerns about avian A/H5N1 influenza, and the recent 2009 influenza A/H1N1 pandemic, more attention has been focused on these respiratory activities as potential sources of infectious aerosols [1]–[7].

Several previous studies have examined various aspects of the airflow dynamics of coughing with human subjects using particle image velocimetry (PIV) [8]–[12], or alternative methods [13], [14], but all of these techniques require some postural and/or physical constraint on the volunteers, e.g. by making them cough into a box or tube or some other specified space, which would not be the case in everyday coughing activities. Most recently, Tang et al. [15] reported horizontal (i.e. x-component resolved) cough propagation distances and velocities using a real-time, non-invasive shadowgraph method. The reported velocities using this technique were in the same range as those reported in these earlier studies.

Sneezing has been much more difficult to investigate, probably because the sneezing reflex is much more difficult to induce on demand for experimental purposes. Two recent studies examined the qualitative effects of sneezing in humans with and without wearing surgical and N95 masks [16], [17], without any quantitative measurements of the sneeze airflows being attempted. One earlier, more physiological study on sneezing in premature newborns suggested that the peak expiratory airflow during sneezing was only 6–7 times higher than that during quiet breathing [18]. This ratio suggests that there may be a discrepancy with the much higher estimates of sneeze velocities reported by Wells [19] of up to 100 m/s, and 50 m/s as has been assumed in some modeling studies [20], though these were described for much older adults.

Breathing generally produces much slower moving exhalation flows than coughing or sneezing, but because we spend more time breathing than coughing or sneezing, it is important to examine and characterize this other human respiratory jet-like exhalation flow as a, perhaps, even more important potential source of infectious aerosols. Relatively few studies have focused on breathing airflow dynamics. Gupta and colleagues performed a series of experiments to characterise the morphology and flow dynamics of nasal and mouth breathing [21], and followed this by computer simulations of how such breath plumes might disseminate and be inhaled in a fully occupied aircraft cabin [22], [23]. Tang et al. [17] used a real-time, non-invasive, shadowgraph method to visualise the airflows produced during nasal and mouth breathing, talking (counting), coughing, laughing and sneezing healthy volunteers, though this was only a qualitative visualisation study without any quantitative assessment being attempted.

The airflow dynamics of coughing were investigated in a previous study using real-time, non-invasive, shadowgraph visualisation [15]. This study applies the same technique to human sneezing and breathing to determine their airflow dynamic parameters. This data may be of use to infection control teams, when planning various interventions to limit the dissemination of airborne infection from human sources.

## Methods

### Imaging Set-up

**Figure 1:**
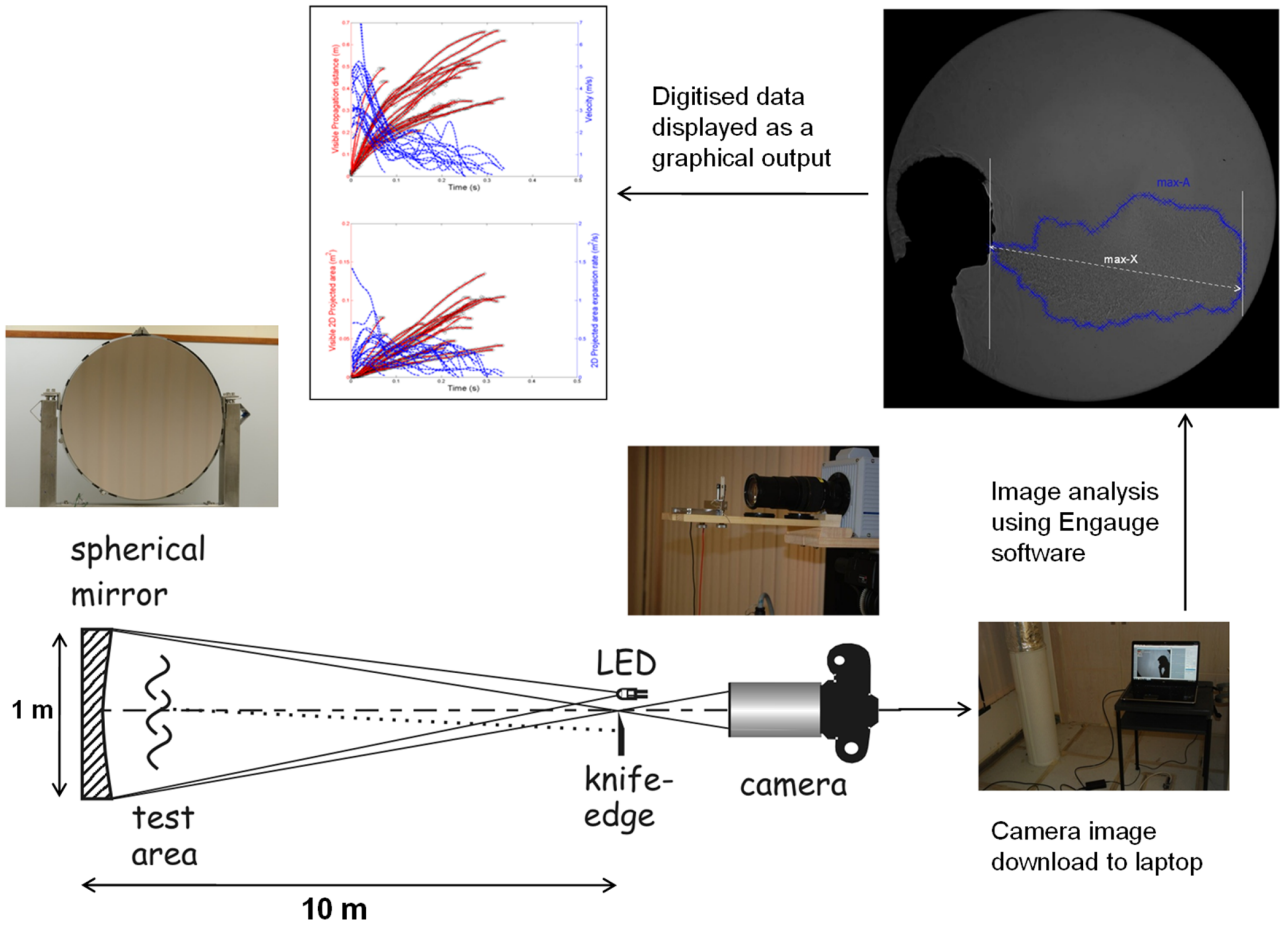
Experimental set-up for the shadowgraph imaging of the human respiratory airflows described in this study (reproduced from Tang et al. 2012).

The shadowgraph imaging technique used in this study has been described and illustrated in detail elsewhere [15], [17]. Briefly, the principle underlying shadowgraph imaging relies on light passing through air of different temperatures, which have differing refractive indices. A large, finely ground, 1-m diameter, spherical, concave, f/5 mirror of astronomical reflector telescope quality (Cosmo Optics Inc., Middletown, NY) was used to reflect a light beam produced by an LED light source placed at the mirror’s centre of curvature (i.e. 10 m from the mirror) through the cooler air (18°C) of the laboratory and the warmer (30–33°C) exhaled air produced by healthy volunteers, to produce the real-time shadowgraph images of their sneezing and breathing airflows for this study. These volunteers were asked to stand approximately 1 m in front of, and just to one side of the mirror in order to maximise the extent of their exhaled flows that would be visible in the mirror (Figure 1).

A high-speed camera (Photron SA1.1, Dynamic Analysis System, Pte Ltd, Singapore) situated just behind the LED light source was used to capture these reflected shadowgraph images at high frame-rates: at 500 frames-per-second (fps) for breathing, and 2000 fps for sneezing. Proprietary, but freely available, software (Photron Fastcam Viewer, PFV Ver.325: http://www.techimaging.com/downloads/) was used to control the high-speed camera remotely from a laptop, which also served to download the image files (in Tagged Image File Format - TIFF) from the camera after every experimental imaging run. The maximum size of the image file recordable by the high-speed camera at any one time was limited to 32 GB (compressed) size, allowing approximately 40 s of filming at 500 fps and 10 s at 2000 fps.

### Human Volunteers

#### Ethics statement

All experiments in this study involving human volunteers were approved by the Domain Specific Review Board of the National University Hospital/National University Health System (DSRB ref no. E/09/024), and all participating volunteers gave both their written and verbal consent.

Twenty healthy volunteers (10 women: mean age 32.2±12.9 years, mean height 1.60±0.07 m, mean weight 53.28±7.23 kg, mean BMI 21.04±3.63; 10 men: mean age 25.3±2.5 years, mean height 1.69±0.06 m, mean weight 63.3±7.00 kg, mean BMI 22.17±1.92) were recruited. These were the same volunteers who performed the coughing experiments as described in Tang et al. [15]. The volunteers were asked to breathe through their nose then through their mouth in front of the mirror. Three cycles of nasal (15–20 seconds) then 3 cycles of mouth breathing (15–20 seconds), were recorded.

For sneezing, none of these initial 20 volunteers were able to sneeze with the approved pepper stimulus. Therefore, another group of 6 volunteers (2 women: mean age 27.5±6.36 years, mean height 1.58±0.04 m, mean weight 47.5±6.36 kg, mean BMI 19.10±1.71; 4 men: mean age 29.25±10.53 years, mean height 1.79±0.06 m, mean weight 76.5±12.18 kg, mean BMI 23.96±3.33) were recruited based on their ability to sneeze in response to the approved pepper stimulus. In addition, several bouts of sneezing from each volunteer were also recorded, until they became tolerant to the pepper stimulus.

Apart from their positioning in front of the mirror, no other constraint was imposed on their body posture or head position.

### Image Analysis

To analyze these images, the digitizing of each volunteer’s breathing or sneezing airflow images was performed by two independent observers, using large (17–19”), flat-screen LCD monitors, at up to 200% magnification, with the observers stepping backwards and forwards between each frame to ensure the continuity of the airflow images, to digitize its airflow boundaries as accurately as possible. By digitising the expanding visible boundary of the exhalation flows generated by sneezing and breathing, frame-by-frame, it was possible to obtain estimates of the evolving propagation distance and area of these airflows over time. From these measurements, additional kinematic parameters, such as the propagation velocity and the two-dimensional (2-D) area expansion rate, were derived.

**Figure 2:**
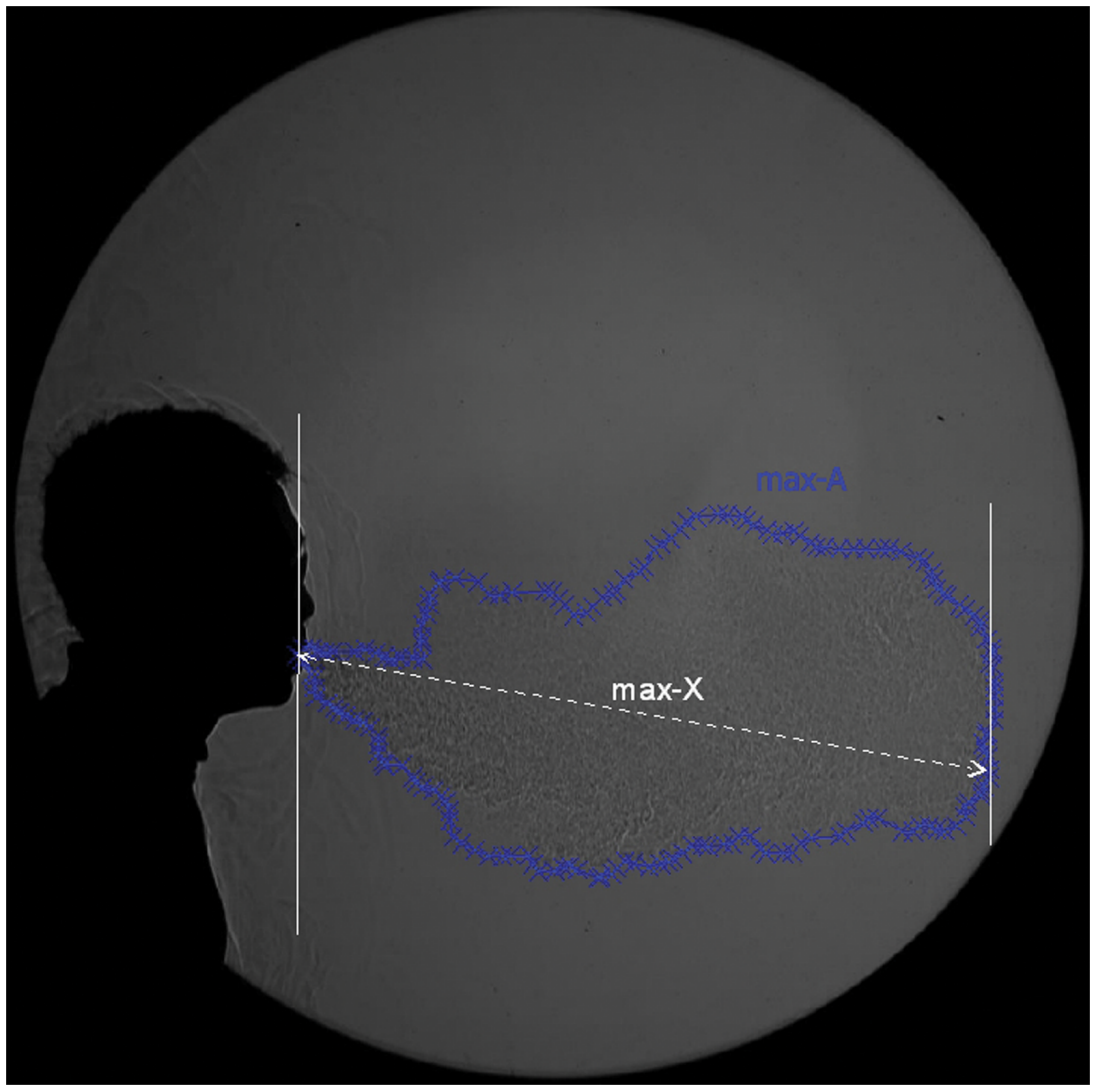
Illustration of the parameters digitised frame-by-frame from the high-speed airflow images captured from each volunteer: the maximum visible propagation distance (max-X) and the maximum visible 2-dimensional (2-D) area (max-A).

A software tool, Engauge Digitizer (freely available from: http://sourceforge.net/projects/digitizer/) was used to convert the visible boundaries of the exhaled airflows to x-y coordinates when manually selected (e.g. by using a computer mouse). An example of the digitising process applied to these images is shown in Figure 2. As these derived velocities were very sensitive to small variations arising from the slight differences in airflow boundary positions as perceived by the different observers, using the raw distance data was thought to be unrepresentative of the true nature of the airflow. To compensate for this, a smoothing algorithm based on weighted moving averages of the distance data was applied to obtain more representative velocity estimates as derived from this raw digitized distance data.

The sneezing or breathing x-y coordinate data was analysed, where the maximum distance (max-X) and 2-D area (max-A) in each frame area of the cough plume was calculated and plotted using custom-written algorithms in C++ and Matlab codes (Matlab v.6.5, MathWorks, Natick, MA http://www.mathworks.com/products/matlab/index.html).

Note that in this analysis, the algorithm to calculate the propagation distance and derived velocities from these images differs slightly from that used to calculate the *horizontal* (i.e. the x-direction resolved component) propagation distance and derived velocities reported in Tang et al. [15]. In this present analysis of the sneezing and breathing, the *maximum* total propagation distance and derived velocities (as opposed to just the horizontal component) was obtained and compared. For comparison purposes, this analysis for maximum propagation distance and derived velocity was also performed for the original cough data from Tang et al. [15], to give the maximum, rather than just the horizontal, x-resolved, values for these parameters.

## Results

**Figure 3:**
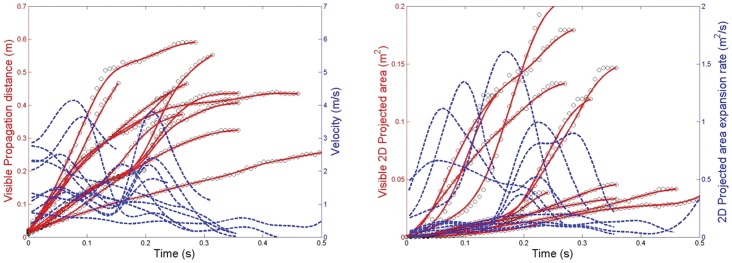
Sneezing airflow parameters. A: Measured visible propagation distances and derived velocities; B: Measured 2-dimensional (2D) areas and derived expansion rates.

The time duration for which reliable measurements could be made using this shadowgraph imaging system (i.e. before the warmer exhaled air had cooled to the same temperature as the ambient laboratory air) was approximately 0.5–2.5 s, depending on the airflow velocities being observed and measured. Within this period, the maximum visible distance over which the sneeze plumes (or puffs) travelled was 0.6 m (Figure 3A), with a maximum derived velocity from this measured distance of 4.5 m/s. The maximum 2-D area of the sneeze plumes (Figure 3B) was 0.2 m^2^, with the corresponding derived parameter, the maximum 2-D area expansion rate equal to 2 m^2^/s.

**Figure 4:**
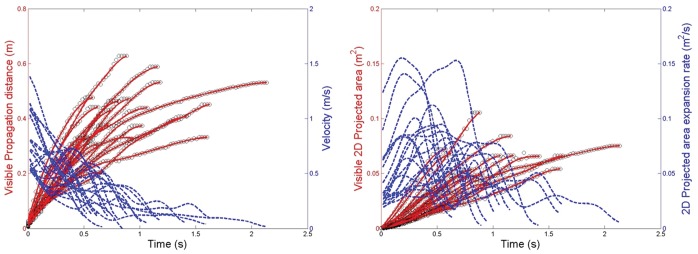
Nasal breathing airflow parameters. A: Measured visible propagation distances and derived velocities; B: Measured 2-dimensional (2D) areas and derived expansion rates.

**Figure 5:**
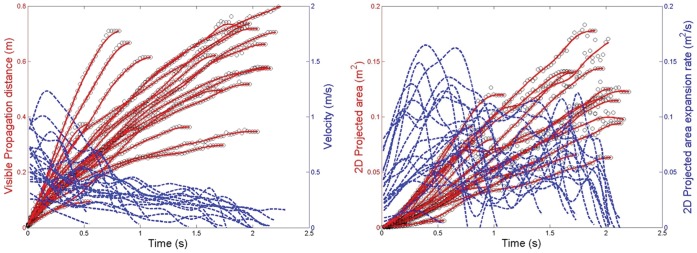
Mouth breathing airflow parameters. A: Measured visible propagation distances and derived velocities; B: Measured 2-dimensional (2D) areas and derived expansion rates.

For the breathing modalities, the maximum visible propagation distance and derived exhalation velocity for nasal breathing were 0.6 m and 1.4 m/s, respectively (Figure 4A), and the maximum 2-D area and expansion rate were 0.11 m^2^ and 0.16 m^2^/s, respectively (Figure 4B). For mouth breathing, the maximum propagation distance and velocity were 0.8 m and 1.3 m/s, respectively (Figure 5A), and the maximum 2-D area and expansion rate were 0.18 m^2^ and 0.17 m^2^/s, respectively (Figures 5B). The profile of these nasal and mouth breathing curves are relatively similar.

**Figure 6:**
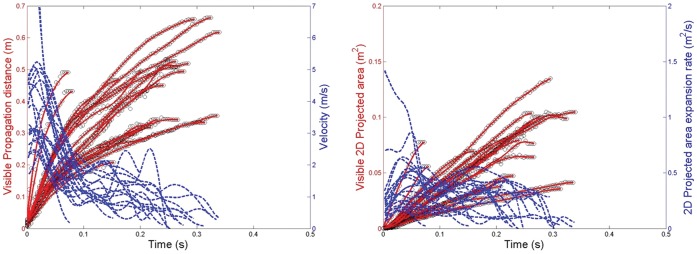
Reanalysed coughing airflow parameters for comparison. A: Measured visible propagation distances and derived velocities; B: Measured 2-dimensional (2D) areas and derived expansion rates.

For the re-analysed cough images, the maximum visible distance over which the cough travelled was 0.7 m, with a *maximum* (i.e. not the purely horizontal, x-component) derived velocity of 5 m/s (with one outlier of approximately 14 m/s), (Figure 6A). The maximum 2-D area for coughing was 0.2 m^2^, with the corresponding derived parameter, the maximum 2-D area expansion rate equal to 1.5 m^2^/s (Figure 6B). The graphs in Figure 6 showing the maximum values of these parameters appear very similar to those shown in Tang et al. [15] (in Figure 3 in [15] – representing the horizontally-resolved, x-component of these parameters) because many of the volunteers’ coughs were in an approximately horizontal direction.

Example video images of all of these respiratory activities (sneezing, breathing and coughing) using this shadowgraph technique have been previously published [17], and readers are referred to this publication for a visual impression of these activities. In particular, for nasal breathing the exhalation plumes for all of the volunteers travelled in a downwards direction, at an angle of 45–60° away from the vertical, whereas for mouth breathing, the exhalation plumes were mainly directed horizontally. From these example video images, surprisingly, neither the nasal nor mouth breathing plumes appeared to be affected by any thermal plume or buoyancy effects within their visible trajectory – indeed, their flows appeared to be driven almost entirely by the exhalation flows from the nose or mouth, with very undisturbed, unidirectional paths.

## Discussion

Perhaps the most striking findings from this study are firstly, that the maximum cough and sneeze velocities are very similar, and secondly, that they are not extremely high - at least in this cohort of human volunteers. As previously discussed in Tang et al. [15], the range of cough velocities reported here are within the range of 1.5–28.8 m/s, as reported elsewhere by several other teams using different techniques [2], [9], [10], [12].

Whilst there are multiple published studies on coughing, there are relatively few estimates of sneeze velocities reported in the literature. Exactly why there is so little comparative data available for sneezing is unclear, though it may be due to the difficulty with inducing the sneeze, which, unlike coughing, is a true reflex [24]. Notably, the few existing estimates are considerably higher than those found in this study. For example, Xie et al. [20] cited a velocity of up to 100 m/s based on earlier estimates by Wells [19], then go on to use estimates of 20–50 m/s for sneeze velocities in a simple physical model of how droplets evaporate and move during various respiratory activities. However, a closer look at Wells’ [19] original estimate reveals that this 100 m/s figure is actually an inference based on “Castleman’s adaptation of Rayleigh’s formulation for droplet formation”, which infers that the velocity required for a moving air stream “sweeping over a liquid surface” to generate droplets of 10 µm diameter is 100 m/s. So this oft-cited figure was only an inference drawn from some basic physical principles applied to a very simplified setting, but not obtained from any direct measurements of airflows generated by actual human sneezes in a much more complex biological environment. It is clear that more data is still needed as the range of velocities cited for sneezing still varies widely, even in this modern era. It should be borne in mind that such values may vary with different measurement methods, as well as the physical attributes, lung capacities and any constrained postures imposed on the human volunteers.

In addition, reported sneeze velocities may vary depending on whether the velocities of the airflows themselves, or droplets that are expelled with them, are measured as an indicator of that sneeze velocity. Early experiments by Jennison and colleagues using strobe lighting and high-speed photography [25], [26] reported sneeze velocities as high as 46 m/s, as measured by the speed with which droplets were expelled by the sneeze. Yet, Jennison [26] acknowledged this distinction by stating: “Depending upon their size and momentum, droplets may move faster, slower, or at the same speed as the air stream in which they are carried.” Therefore, it may well be this distinction that has led to such wide variability in the reported sneeze velocities. Using the speed of the expelled droplets as a measure of the sneeze velocity then begs the natural follow-up question of which droplet size should then be used as a measure of sneeze velocity? Wells’ [19] inferred estimate of 100 m/s for the sneeze velocity seems to assume that this droplet size is 10 µm in diameter. Again, more experimental studies (as opposed to theoretical inferences) are clearly needed, particularly into the distribution of droplet sizes and their velocities as produced by human sneezes, to answer these questions more accurately.

With this shadowgraph method, the fact that the maximum cough velocities obtained using this approach are comparable with other published experimental methods, does indicate that the same approach used for the measurement of sneeze velocities should also produce fairly accurate estimates of the true values. The greater apparent variation and difference in overall curve profiles in the propagation distances and derived velocities with the sneezes, as compared to the coughs (compare Figures 3 and 6), is most likely due to the fewer images that were available for analysis as there were fewer volunteers in the sneeze cohort. It is acknowledged that higher sneeze velocities may well be possible with other volunteers, and that further studies are encouraged to explore and better characterize this variable.

Note that for most of the volunteers, the maximum sneeze velocities are generally not reached until some short time after the airflow leaves the mouth (see Figure 3). This probably represents the acceleration of the expelled air in the early expiration phase of the sneeze, after the initial inspiration stage of the sneeze reflex, which is well recognized [24]. This initial inspiration stage was also recognized much earlier by Jennison [26], as he stated: “A sneeze consists of two stages – a sudden inspiration, followed by a forcible expiration.” In addition, the changing shape of the mouth and position of the tongue may well affect the exit velocity of the air during the sneeze. A similar explanation to this was suggested by Tang et al. [15] for very similar appearances of the cough distance-velocity curves in that study. The shape of these curves remains similar, as seen in Figure 6, which shows the same cough dataset re-analyzed to give the maximum (not just the x-resolved) cough velocities. The results in Figures 3 and 6 demonstrate that the maximum exit velocities of 4–5 m/s for coughing and sneezing, at least in this cohort of healthy young volunteers, were not very different, and far less than idealized 100 m/s inferred velocity for sneezing by Wells [19].

For the nasal and mouth breathing, relatively little data has been published so the results from this study will contribute to this. The exhalation airflow profiles for both nasal and mouth breathing are quite similar, being mainly conical and differing only in their relative direction, with similar propagation distances and airflow velocities. The variations in the airflow velocities of the nasal and mouth breathing exhalation flows can be related to the variation of airflow rates during expiration, which are due to a combination of lung tissue and diaphragmatic elasticity and recoil and elasticity that have been well-documented [27]. A study on the airflow dynamics of breathing by Gupta et al. [21] in human volunteers was performed on smokers, which may not be necessarily representative of healthy volunteers. Unfortunately, they used units of airflow rate (litres per second) which are difficult to convert to comparable airflow velocities (in m/s). Finally, the ratios of the velocities of the sneeze to those of the nasal and mouth breathing in this cohort of healthy, young volunteers is 3–4, which is not so dissimilar to that described by Javorka et al. [18] in premature newborns with a ratio of 6–7, and also suggests that this sneezing:breathing ratio may well be decreased in older, larger adult bodies.

This shadowgraph technique has some limitations in that the maximum propagation distances can only be observed whilst there remains a temperature difference between the exhaled and ambient laboratory air. This may result in some underestimation of the maximum dissemination distances for some of these human respiratory activities. However, a minimum ‘observable’ dissemination distance has been given here, which is still of use to infection control teams. The maximum exit velocities for sneezing, breathing and coughing all occur within a time-frame for which the airflows are clearly visible, so these real-time velocity estimates should be relatively accurate. Ideally, these human voluntary ‘jet-like’ airflows, as imaged by this shadowgraph technique, should be compared to more controlled, idealized jet airflows to better understand and characterize this experimental method for visualizing such phenomena. However, within the more practical context of everyday, clinical patient infection control situations, these measurements from these human volunteers should still be useful.

In summary, this study adds new data using a new, non-invasive, visualization approach to the airflow dynamics of sneezing and breathing in healthy human volunteers. It also makes a direct comparison between maximum cough and sneeze velocities using this shadowgraph method, which, surprisingly, shows them to be firstly, quite similar in speed, and secondly, that this speed is not extremely high, as has been inferred in some older estimates of sneeze velocity.
